# Diagnosis and treatment of retroperitoneal fibrosis: A case report

**DOI:** 10.3892/etm.2013.943

**Published:** 2013-02-01

**Authors:** BOLUO LIANG, ZHUO YIN, QIONG GUO, YONGBAO WEI, LEI LIU, JINRUI YANG

**Affiliations:** 1 Department of Urology, The Second Xiangya Hospital, Central South University, Changsha 410011, P.R. China; 2 Department of Urology, The Third Hospital Of Changsha, Changsha 410000, P.R. China; 3 Department of Urology, Weihai Municipal Hospital, Weihai 264200, P.R. China

**Keywords:** diagnosis, treatment, retroperitoneal fibrosis

## Abstract

Retroperitoneal fibrosis (RPF) is a rare disease of unclear etiology, which is characterized by a chronic non-specific inflammation of the retroperitoneum. The present study reports the case of a 36-year-old male with a 3-month history of lower right abdominal pain (intermittent) and weight loss (5 kg). A mass was identified that covered the surface of the abdominal aorta and the inferior vena cava near the right renal hilum. Three shots with an automated gun were employed to biopsy the mass. The patient began taking prednisone one month subsequent to the surgery at a dose of 10 mg, three times a day once a month and at continuously reducing doses for 1 year. CT scans showed that the retroperitoneal mass decreased in size with the progression of the treatment and that the mass had almost disappeared on the final month’s MRI scan. In conclusion, the diagnosis of retroperitoneal fibrosis is an effective process that excludes the other diagnoses for the lesion. A biopsy of the mass is a necessity for the final stage of diagnosis and is supported by the response to the steroid treatment.

## Introduction

Retroperitoneal fibrosis (RPF) is a rare disease of unclear etiology, which was first described by Albarran in 1905 (1). Recently, there have been more cases of this disease reported (2,3). RPF is characterized by a chronic non-specific inflammation of the retroperitoneum, which may entrap and obstruct retroperitoneal structures, particularly the ureters.

Patients with RPF show non-specific clinical symptoms, including poorly localized back pain, general malaise, weight loss, anemia, features of renal failure and occasionally, mild fever (4–7). In addition, RPF is difficult to detect. In the present case report, a patient that had been suffering from abdominal pain and weight loss for three months was primarily diagnosed with lumbar and back myofascitis. However, traditional treatments had no effect on the patient, therefore, a further examination was performed and a biopsy of the mass was employed. Prednisone was administered to the patient and the retroperitoneal mass decreased in size and finally disappeared.

## Case report

**Figure 1:**
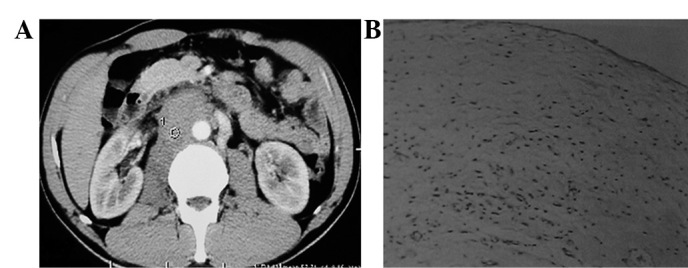
(A) CT scan of the abdomen showing the retroperitoneal mass on the plane of the right renal vein. The inferior vena cava was surrounded by the mass. (B) Mass biopsy with retroperitoneal laparoscopy showing fiber and fat tissues infiltrated by inflammatory cells.

A 36-year-old male was admitted to the Second Xiangya Hospital, Central South University (Changsha, China) with a 3-month history of lower right abdominal pain (intermittent) and weight loss (5 kg). The patient had no previous history of drug use for specific medical conditions or lymphoma and no drug or food allergies. A physical examination showed that the patient’s abdomen was soft and flat with normal bowel sounds. No pain sensation was detected in the bilateral renal area and the superficial lymph nodes were not palpable. The patient’s fecal occult blood test results were negative and the urinalysis result was normal. The complete blood cell count showed normochromic normocytic anemia with a hematocrit level of 32.2% (normal value, 37–51%). The blood urea nitrogen level was 3.83 mmol/l (normal value, 2.9–7.14 mmol/l) and the serum creatinine was 71.9 *μ*mol/l (normal value, 40–133 *μ*mol/l). The plasma β2-microglobulin level was 3.66 mg/l (normal value, 1–3 mg/l), the serum ferritin was 375.26 ng/ml (normal value, 21.8–274.66 ng/ml) and the C-reactive protein was 72 mm/l (male, 0–15 mm/l). The human leucocyte antigen-B27 and tuberculosis antigen analysis results were negative. The antistreptolysin O test and rheumatoid factors were also negative. The tumor marker levels, including those of carcinoembryonic antigen (CEA), CA19-9, CA125, lactate dehydrogenase and alpha-fetoprotein (AFP) were within normal ranges. The results of the other biochemical screening and electrolyte tests were all within normal limits. An infrared thermal imaging examination showed lumbar and back myofascitis. Abdominal ultrasonography, gastrointestinal endoscopy and colonoscopy showed no noteworthy findings. A CT scan of the abdomen revealed a retroperitoneal mass (∼7×2 cm) on the plane of the right renal vein (Fig. 1A) with no signs of hydronephrosis or ureteral dilation. A retroperitoneal mass biopsy was then performed laparoscopically. An adhesion was identified between the right upper ureter and the inferior vena cava during the surgery. The mass covered the surface of the abdominal aorta and the inferior vena cava near the right renal hilum. Three shots with an automated biopsy gun were employed to biopsy the mass. The results showed that the biopsy specimen was made up of fiber and fat tissues which were infiltrated by inflammatory cells (Fig. 1B).

**Figure 2:**
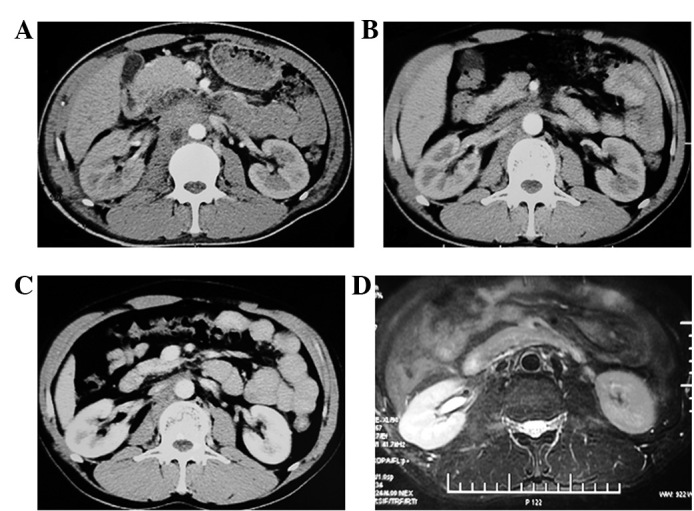
CT scan images and MRI showing the stages of the physiological changes of the abdomen following the treatments of prednisone. (A) The first month of the treatment. The CT scan showed that the mass was the same size, but the clinical symptoms, including the back pain, were reduced. (B) The third month of the treatment of prednisone. The CT scan showed that the mass was much smaller than in the first month. (C) The sixth month of the treatment of prednisone. The CT scan showed that the border of surrounding tissues was clear and that the mass was further reduced in size. The clinical symptoms disappeared. (D) The last month of the treatment of prednisone. MRI showed that the mass had almost disappeared and that the outlines of the abdominal aorta and inferior vena cava were clear.

The patient began taking prednisone one month subsequent to the surgery at a dose of 10 mg, three times a day for one month. The dose was reduced to 25 mg once each day after one month and then reduced by 5 mg each day. A dose of 5 mg was maintained lasting for approximately two months until August 2011 and then was reduced to 1 mg once each day for another month. Therefore, in total, the prednisone was continuously taken by the patient for 1 year. During this treatment, the patient underwent a CT scan of the abdomen every three or six months. The images from the CT scans showed that the retroperitoneal mass was decreasing in size with the progression of the treatment (Fig. 2A, B and C). The mass had almost disappeared on the final month’s MRI scan in August (Fig. 2D). Prior written and informed consent were obtained from every patient and the study was approved by the ethics review board of Central South University.

## Discussion

RPF is a rare disease of unclear etiology; however, the clinical manifestations are non-specific in the early stages of its course. It is important for researchers to create enough awareness of this disease. The lesions often begin with aortic associations, gradually ending as associated with the aorta, inferior vena cava and ureter and also involved with the mesenteric and renal arteries (8). The disease may cause obstructions of certain structures, including the mesenteric artery, duodenal choledochal duct and inferior vena cava.

Data accumulated from China reveal that RPF occurs mainly in males in their fifties and sixties, with the male-to-female ratio being ∼1.9:1 (9). The early symptoms are non-specific and an accurate diagnosis is often achieved only subsequent to urological obstruction or the occurrence of renal failure. The majority of patients feel pain in the lower back, flank and/or abdomen. These are the most obvious symptoms and the pain is dull and non-colicky in the beginning, becoming more severe with the progress of the disease. Other symptoms that are quite frequent include weight loss, anorexia, nausea, vomiting, claudication, ureteral colic and hematuria. Symptoms including fever, testicular pain, abdominal angina, intermittent claudication, edema, scrotal effusion and Raynaud’s syndrome are quite rare. Meanwhile, certain symptoms, including various degrees of ureteral obstruction, hydronephrosis and renal failure, are the earliest and most common clinical manifestations. Although a number of scientific journals devoted to RPF are present in the literature, there is no accepted diagnostic or therapeutic strategy for this disease. However, there are several therapeutic strategies which have been proven to be effective. It is commonly noted in the literature that patients with non-malignant RPF are treated primarily with steroids or with a combination of azathioprine (2 mg/kg) and steroids (10–12).

In the present study, the patient’s physical examination was normal, with the exception of the presence of back pain. The mass was located on the plane of the right renal vein with no signs of hydronephrosis or ureteral dilation on the CT scan. The back pain became more serious with the progression of the therapy. In addition, the aggravation of the pain was accompanied with side effects, including vomiting and nausea. The mass was observed to be closely adhered to the inferior vena cava during the surgery and their separation was not possible. The results of the biopsy showed that the specimen was made of fiber and fat tissues infiltrated by inflammatory cells, therefore, a diagnosis of malignancy or abscess was excluded. The biochemical tests on the serum for conditions, including autoimmune system diseases and lymphoma, were negative. Therefore, the patient was treated with prednosione and the pain symptoms and the retroperitoneal mass almost disappeared subsequent to one year of treatment. Afterwards, a diagnosis of early stage retroperitoneal fibrosis was established.

In conclusion, the diagnosis of retroperitoneal fibrosis is an effective process that excludes the other diagnoses for the lesion. A biopsy of the mass is a necessity for the final stage of diagnosis and is supported by the response to the steroid treatment. CT scans or MRI are effective examination tools, which help researchers to diagnose and monitor the progress of the disease.
